# Medical Missions to China

**Published:** 1837-10

**Authors:** 


### MEDICAL MISSIONS TO CHINA.

By a notice inserted in the Advertising Sheet annexed to this Journal, it will be observed that the London Missionary Society is desirous of finding medical men adapted to execute its benevolent designs for the improvement of the vast and interesting population of China. To candidates properly qualified, such an undertaking must be highly attractive. In a scientific point of view, China presents a field of observation of great variety and extent, and hitherto but imperfectly explored. With slight exceptions, the state of medical knowledge and practice in this ancient country is extremely low and defective; and, notwithstanding their inordinate national vanity, many of the inhabitants are beginning to recognize the superiority of Europeans in this, as well as in many other departments. A competent endowment of medical science, and more especially a talent for operative and ophthalmic surgery, would be a sure passport to popularity and reputation under such circumstances; and we can hardly imagine a situation more calculated to excite and gratify the honorable ambition and philanthropic feelings of generous and adventurous youth.

